# Liver abscess and bacteremia caused by *Streptococcus constellatus* with suspected ileocecal valve lesion as the entry point

**DOI:** 10.1016/j.idcr.2025.e02485

**Published:** 2025-12-29

**Authors:** Yuma Takeda, Takaaki Kobayashi, Nicholas Van Sickels, Akihito Yoshida

**Affiliations:** a Department of General Internal Medicine, Kameda Medical Center, Chiba, Japan; b Department of Internal Medicine, University of Kentucky, Lexington, KY, USA

**Keywords:** *Streptococcus constellatus*, Pyogenic liver abscess

## Abstract

Pyogenic liver abscess (PLA) is a rare but potentially life-threatening condition. *Streptococcus constellatus*, part of the *Streptococcus anginosus* group, is an uncommon causative agent of PLA, though its incidence has been increasingly reported. We present the case of a 68-year-old man with a history of hypertension, type 2 diabetes, and dyslipidemia, who was admitted after a traffic accident. Medical evaluation revealed bacteremia and liver abscesses caused by *S. constellatus*, and the patient was treated successfully with drainage and antibiotics. While the patient denied gastrointestinal symptoms, a colonoscopy was performed to investigate a possible portal of entry for the organism, which revealed an inflammatory lesion at the ileocecal valve. This case underscores the importance of colonoscopy in identifying potential sources of infection in cryptogenic PLA and highlights the need for thorough evaluation of gastrointestinal lesions in patients with *S. constellatus* bacteremia. The patient's loss of consciousness during the traffic accident was attributed to sepsis, reinforcing the critical role of comprehensive internal investigations in trauma patients with unexplained symptoms.

## Introduction

The most common bacteria isolated from PLA are *Escherichia coli*, *Klebsiella pneumoniae*, *Enterococcus faecalis*, and anaerobes such as *Bacteroides fragilis* [1]. The *Streptococcus anginosus* group (SAG), also known as the *Streptococcus milleri* group, comprises three species: *S. anginosus, S. intermedius*, and *Streptococcus constellatus* [2]. These Gram-positive, catalase-negative cocci are notorious for their ability to form abscesses and routinely colonize the human oropharyngeal and gastrointestinal tracts. Of the SAG species, *S. constellatus* is most implicated in intra-abdominal and perirectal abscesses, which are often polymicrobial. When encountered in blood cultures, they are rarely considered contaminants [3]. In this report, we present a case of *S. constellatus* bacteremia and liver abscess in a 68-year-old Japanese man without gastrointestinal symptoms, who was found to have an ileocecal lesion on colonoscopy.

## Case

A 68-year-old man with a medical history of cardiovascular and cerebrovascular disease, prostate cancer status post radical prostatectomy, and prior scrub typhus presented to the emergency department (ED) after a motor vehicle accident precipitated by loss of consciousness (LOC). He had been in his usual state of health before the event and denied fever, chills, abdominal pain, or changes in bowel habits. While driving, he suddenly experienced dizziness and loss of consciousness, resulting in a single-vehicle collision. The only physical injury was to his knee, which hit the dashboard upon impact. On arrival, his temperature was 37.3°C, his heart rate was 90 beats/min, and blood pressure was 170/100 mmHg. Physical examination revealed a multi-centimeter laceration over the right knee, without other notable findings. Initial laboratory results are summarized in Table 1. Computed tomography of the head, chest radiography, and abdominal radiography were unremarkable, as were electrocardiography and plain radiographs of the knee. He was deemed clinically stable, the knee laceration was sutured, and he was discharged home. Given the low-grade fever and elevated C-reactive protein and liver function tests, two sets (four bottles) of blood cultures and urine culture were obtained. He was advised to return to the ED if his condition worsened.

**Table 1:** Laboratory test results.

**Variable**	**Unit**	**Reference range**	**Initial visit**	**Day 5** **PTAD placement**	**Day 43** **The last test during admission**
WBC	/μL	3300–8600	18,300	11,000	6,800
Neutrophils	%	42.4–75.0	91.6	82.9	-
Hemoglobin	g/dL	13.5–17.6	13.2	11.5	11.7
CRP	mg/L	< 1.4	171.0	105.7	1.8
AST	U/L	13–30	85	83	13
ALT	U/L	10–42	54	72	15
LD	U/L	124–222	474	204	147
ALP	U/L	38–113	123	164	125
γ-GT	U/L	13–64	33	38	21
Total bilirubin	mg/dL	0.4–1.5	0.8	0.4	0.5
BUN	mg/dL	8–20	14	12	9
Creatinine	mg/dL	0.65–1.07	0.77	0.69	0.90

PTAD: percutaneous transhepatic abscess drainage, WBC: White blood cell, CRP: C-reactive protein, AST: Aspartate aminotransferase, ALT: Alanine aminotransferase, LD: Lactate dehydrogenase, ALP: Alkaline phosphatase, γ-GT: Gamma-glutamyltransferase, BUN: Blood urea nitrogen

**Fig. 1:**
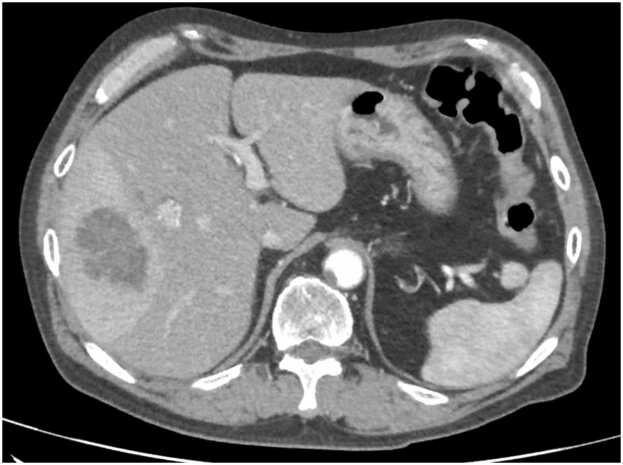
Contrast-enhanced computed tomography of the abdomen. Multiple lobulated low-attenuation areas are observed in the liver, with wedge-shaped areas of early enhancement surrounding them in the arterial phase.

The following day, he developed a subjective fever and returned to the ED. A more detailed history revealed no recent antibiotic use, hospitalizations, or the presence of indwelling medical devices. He reported smoking approximately 20 cigarettes per day and consuming about 50 g of alcohol daily, with no use of illicit substances. His medications included clenbuterol hydrochloride, nifedipine, and vibegron. On presentation, he was afebrile (36.8°C), mildly tachycardic (92 beats/minute), and normotensive (111/73 mmHg). Physical examination was notable only for the sutured right knee laceration. Laboratory studies showed persistent leukocytosis (WBC 16800/μL; 91.6 % neutrophils), a markedly elevated C-reactive protein (237.5 mg/L), and ongoing liver enzyme abnormalities. Contrast-enhanced CT of the abdomen (Fig. 1) demonstrated multiple round lesions with central hypoattenuation in the liver, consistent with hepatic abscesses.

Given this new finding and his recent LOC, magnetic resonance imaging (MRI) of the brain was obtained, revealing small, acute ischemic infarcts in the right anterior central gyrus and right temporal lobe, which were not considered significant enough to account for the initial episode of LOC. He was admitted for further evaluation and empirically started on intravenous ampicillin/sulbactam 3 g every 6 h for presumed bacterial liver abscess. Abdominal MRI was performed to assess for biliary tract infection or structural abnormality, which was unremarkable.

Initial blood cultures grew *Streptococcus constellatus* in one aerobic and one anaerobic bottles from the same set, confirmed by Matrix-Assisted Laser Desorption/Ionization Time-of-Flight Mass Spectrometry (MALDI-TOF MS). The urine culture obtained concurrently yielded no bacterial growth. His history of multiple cerebral infarctions raised concerns for infectious cerebral embolisms secondary to infective endocarditis (IE). Pending a complete evaluation for IE, antibiotics were changed to intravenous ceftriaxone 2 g every 24 h plus oral metronidazole 500 mg twice daily to ensure adequate central nervous system penetration. Transesophageal echocardiography was performed and was negative for valvular vegetations. Additionally, repeat blood cultures were negative. Given the low probability of IE-related embolisms, antibiotics were changed back to ampicillin/sulbactam. On hospital day 7, percutaneous transhepatic abscess drainage (PTAD) was performed. Gram stain and culture from the abscess were negative, likely due to preceding antibiotic exposure for five days. 16S ribosomal RNA gene sequencing analysis was not performed. Ampicillin/sulbactam was continued to maintain anaerobic coverage, as *S. constellatus* liver abscesses are frequently polymicrobial and the negative aspiration culture obtained after broad-spectrum antibiotic exposure did not exclude concomitant anaerobic infection [4]. On hospital day 27, the drainage tube was removed.

**Fig. 2:**
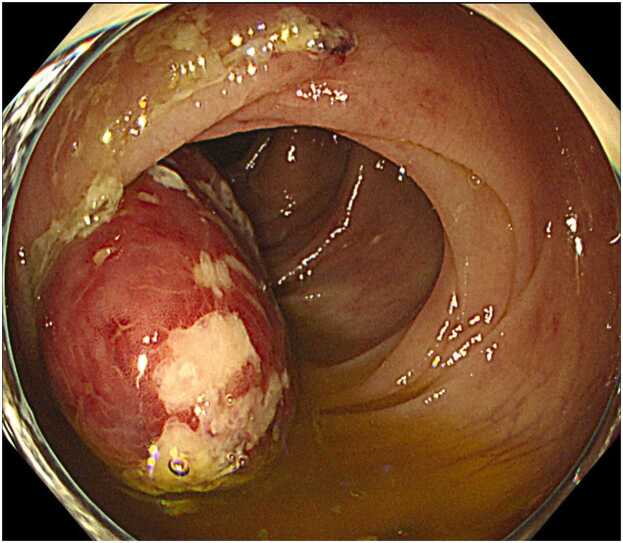
Colonoscopy finding. An erythematous polyp extending toward the proximal side of the ileocecal valve is observed, with partial surface erosion. A biopsy was performed at two sites within the lesion.

**Fig. 3:**
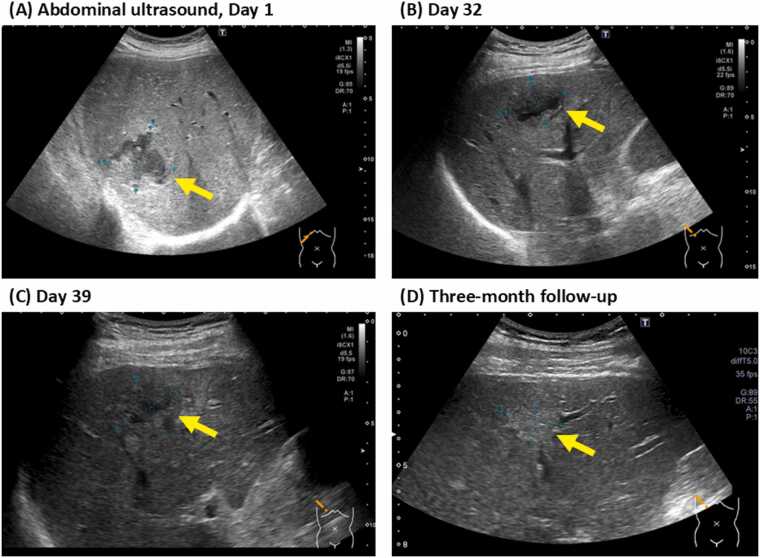
Serial abdominal ultrasound findings of the liver abscesses during antibiotic treatment, (a) On day 1, the largest abscess measured 50.4 × 38.8 mm. (b) On day 32, the abscess remained at 40.6 × 27.2 mm. (c) (C) On day 39, the abscess had decreased in size to 33.7 × 29.7 mm, following continued intravenous antibiotic therapy. (d) (D) At the three-month follow-up, abscess size reduction to 21.6 × 12.0 mm with residual scarring was confirmed.

A dental and oral examination did not reveal any potential sources of infection. The isolation of *S. constellatus* from blood cultures in conjunction with hepatic abscesses prompted a gastrointestinal evaluation. Colonoscopy (Fig. 2) identified an inflammatory-appearing lesion at the ileocecal valve, which was biopsied. Pathologic examination of the specimen, following endoscopic mucosal resection, demonstrated acute colitis with abscess formation, considered the most likely portal of entry for the bacteremia. Cultures of the resected tissue were negative for bacteria, fungi, and mycobacteria. The small cerebral infarctions seen on MRI were thought to be sequelae of sepsis, not IE. The size of the abscess was monitored by serial abdominal ultrasonography. The largest abscess measured 57.5 × 53.6 mm on day 1 (Fig. 3A). A repeat ultrasound on day 32 showed a reduction in abscess size to 40.6 × 27.2 mm (Fig. 3B), but intravenous antibiotic therapy was continued throughout hospitalization. Confirming a further decrease in size to 33.7 × 29.7 mm on day 39 (Fig. 3C), the patient was discharged on hospital day 48 with oral amoxicillin–clavulanate (500 mg/125 mg) four times daily. At a three-month follow-up, antibiotics were discontinued as repeat abdominal ultrasonography confirmed resolution of the liver abscess with residual scarring (Fig. 3D).

## Discussion

The annual incidence of PLA is estimated to range from 2.3 to 3.6 cases per 100,000 people worldwide, with substantially higher rates reported in East Asian countries, reaching up to 17.59 per 100,000 [1], [2]. Risk factors include liver transplantation, diabetes, alcohol use, and malignancy [5]. Although uncommon, PLA is a potentially life-threatening condition. Advances in treatment have reduced mortality rates, with recent studies reporting a reduction in mortality from 6 % to 19–5.6 % [6]. The most common underlying causes are biliary tract disease or dissemination via the arterial or portal vein systems. Other etiologies include trauma, tumor-associated architectural changes in the liver, or perforation of adjacent organs [7].

Symptoms can vary, with a large cohort reporting fever in 89.6 % of patients, right upper quadrant pain and tenderness in 72.2 %, and chills in 69.0 %. Notably, only 43 % of patients exhibit the combined triad of fever, right upper quadrant pain, and elevated alkaline phosphatase levels [1]. Common laboratory abnormalities include hypoalbuminemia, leukocytosis, and elevated alkaline phosphatase levels.

Since the clinical presentation of PLA is generally nonspecific, imaging modalities such as ultrasonography and contrast-enhanced CT play crucial roles in diagnosis. Fine needle aspiration for culture remains the gold standard for identifying causative pathogens.

In the case presented here, colonoscopy revealed an abscess located at the ileocecal valve. Common pathogens associated with ileocecal infection include *Salmonella*, *Campylobacter*, and *Yersinia* species [3]. *Mycobacterium tuberculosis* may also affect ileocecal region due to its rich gut associated lymphoid tissue [8]. Although *Streptococcus* species are not typically associated with ileocolitis, they may enter the bloodstream through mucosal damage caused by other etiologies, including infection, ischemia and tumor.

In general, liver abscess cultures yield a mixture of enteric Gram-positive, Gram-negative, and anaerobic bacteria [1]. In this patient, aspirates from the liver lesions were sterile, likely because drainage was performed after the initiation of antimicrobial therapy. However, blood cultures obtained prior to treatment grew *Streptococcus constellatus*, supporting its role as the causative organism in this case of PLA. Although *S. constellatus* was isolated from only one of two blood culture sets, organisms within the *Streptococcus anginosus* group are rarely contaminants and are strongly associated with deep-seated abscess formation, including PLA [4].

*S. constellatus* belongs to the SAG along with *S. anginosus* and *S. intermedius*. Most *S. constellatus* strains carry Lancefield group F antigens or are not groupable. The majority of Lancefield group F strains exhibit beta-hemolysis [9]. SAG species are part of the normal flora of the oropharyngeal, gastrointestinal and reproductive tracts [10]. They are frequently associated with abscess formation in these sites as well as others – including skin, soft tissue, and brain. Among the SAG species, *S. constellatus* and *S. intermedius* are more likely than *S. anginosus* to cause deep-seated abscesses [10]. Although *S. constellatus* is a relatively rare cause of liver abscess, the number of reported cases is increasing. In addition to abscess formation, members of the SAG represent 3–15 % of streptococcal isolates from patients with endocarditis [11], [12]. Their ability to adhere to buccal epithelial cells and to bind to fibronectin may contribute to their pathogenicity [4].

Optimal treatment of abscesses generally requires both antimicrobial therapy and drainage. *S. constellatus* is typically susceptible to penicillin, though initial therapy should be broad and include Gram-negative and anaerobic coverage, as most infections associated with this species tend to be polymicrobial. When cultures and susceptibilities result, antimicrobials can be narrowed accordingly [13]. In this case, the patient was successfully treated with drainage and antibiotics, including ampicillin/sulbactam and amoxicillin/clavulanate.

Although there is no consensus on the routine use of colonoscopy in PLA cases, a recent retrospective cohort study found that colorectal cancer was significantly more common among PLA patients than among controls. Additionally, this association was not observed in patients with cryptogenic PLA, suggesting that colorectal cancer screening may be beneficial in such cases [14]. Despite this, only 3.8 % of PLA cases undergo colonoscopy [1]. As a result, it is possible that many underlying lower gastrointestinal lesions go undetected.

Identifying the underlying cause of liver abscess is crucial to prevent recurrence. In this case, colonoscopy played a key role in identifying the portal of entry for *S. constellatus*. A literature search using PubMed® with the terms "([liver abscess] OR [hepatic abscess]) AND (Streptococcus constellatus)" yielded 30 results. After excluding articles not written in English or Japanese and those unrelated to liver abscesses caused by *S. constellatus*, 20 relevant reports were reviewed. Of these cases, 10 identified lower gastrointestinal sources of entry, including diverticulitis in five cases, miscellaneous inflammatory conditions in two, and neoplasms in two. Upper gastrointestinal and oral sources were reported in three and one case, respectively (Table 2). Interestingly, unlike typical PLA cases, biliary tract infection has not been reported as an entry point in PLA caused by *S. constellatus*, underscoring the importance of considering colonoscopy to identify the portal of entry for PLA caused by *S. constellatus* [21], [33].

**Table 2:** Summary of patients with 20 cases of liver abscess due to Streptococcus constellatus identified through a PubMed® search.

Rererence	Age	Sex	PMH	Estimated portal of entry	Blood culture	Abscess culture	Drainage	Antibiotics
Badar F,[15]	39	M	DM, diverticulitis	Periapical abscess of the left mandibular central incisor	*S. constellatus*	*S. constellatus*	Performed	PIPC/TAZ
Miike T, [16]	49	F	Not documented	Ileal tumor	Not documented	*S. constellatus*	Not documented	SBT/CPZ + VCM -> PCG + CLDM -> MEPN + CLDM
Gharib SD, [17]	37	M	None	Fish born penetrating the duodenum	*S. constellatus*	*S. constellatus*	Performed	DOXY -> CTRX
Koay S,[18]	52	M	None	Diverticulitis	*S. constellatus*	Not documented	Not documented	AMPC + GM -> AMPC
Harnik IG, [19]	68	M	DM, HTN, HL	Polypectomy of a colon adenocarcinoma	Not documented	*S. constellatus*	Not documented	not documented
Samra GS, [20]	35	F	obesity	Not documented	*S. constellatus*	*S. constellatus*	Performed	PIPC/TAZ + VCM + MNZ -> CTRX + MNZ -> CFDN
Kokayi A, [21]	36	M	None	Inflamed terminal ileum probably caused by complicated appendicitis	Not documented	*S. constellatus*	Performed	CFX + MNZ + GM -> AMPC/CVA
Akuzawa N, [22]	69	M	HTN	Colon diverticulitis at hepatic flexture	*S. constellatus*	*S. constellatus*	Performed	MEPM -> ABPC + GM
Riaz MF, [23]	41	M	asthma, UTI, erectile dysfunction and diverticulosis	Sigmoid diverticulitis	*S. constellatus*	*S. constellatus*	Performed	LVFX + MNZ
Kim DH, [24]	47	M	None	Not documented	Not documented	*S. constellatus*	Performed	CTRX + CPFX + MNZ
Ward TE, [25]	29	F	None	Acute appendicitis	Not documented	*S. constellatus, Bacteroides fragilis, and Bacteroides ovatus*	Performed	CTRX + MNZ
Wong Y, [26]	81	M	HTN	Gastric adenocarcinoma	*S. constellatus*	Not documented	Performed	MNZ + flomoxef -> cefotaxime 2 g ivd q6h and ampicillin
Rodrigues ALS, [27]	23	M	None	Not documented	Not documented	*S. constellatus*	Performed	CTRX + MNZ + ABPC -> CPFX
Navarrete D, [28]	54	M	HTN	Sigmoid diverticulitis	*S. constellatus*	*S. constellatus*	Performed	CTRX + MNZ
Mohanty S, [29]	43	M	None	Not documented	*S. constellatus*	not documented	Performed	CTRX
Nahidi SM, [30]	72	F	Diverticulitis, transient ischemic attack, CAD, fibromyalgia, spinal fusion surgery and anxiety disorder	Diverticulitis	*S. constellatus*	*S. constellatus*	Performed	CTRX + MNZ -> MEPM
Dsouza R, [31]	26	M	None	Not documented	*S. constellatus*	*S. constellatus*	Performed	crystalline penicillin for 4 weeks
Khan MZ, [32]	78	M	HTN, stroke, BPH, vascular dementia and myocardial infarction	Not documented	*S. constellatus*	*S. constellatus*	Performed	Cefepime + Ampicillin/Sulbactam + Metronidazole -> Clindamycin + Ceftriaxone
Chessa S, [33]	80	M	Hypertensive heart disease	Gastrointestinal stromal tumor in the jejunum	*S. constellatus*	not documented	Not performed	PIPC/TAZ -> PIPC/TAZ + TEIC
Govea M, [34]	19	F	None	Not documented	Negative	*S. constellatus*	Performed	PIPC/TAZ

M: Male

F: Female

PMH: Past Medical History

HTN: Hypertension

DM: Diabetes Mellitus

HL: Hyperlipidemia

BPH: Benign Prostate Hyperplasia

CAD: Coronary Artery Disease

UTI: Urinary Tract Infection

PIPC: Piperacillin

TAZ: Tazobactam

CPZ: Cefoperazone

VCM: Vancomycin

PCG: Penicillin G

CLDM: Clindamycin

MEPM: Meropenem

DOXY: Doxycycline

CTRX: Ceftriaxone

AMPC: Amoxicillin

GM: Gentamicin

MNZ: Metronidazole

CFDN: Cefdinir

CFX: Cefalexin

CVA: Clavulanic acid

LVFX: Levofloxacin

ABPC: Ampicillin

TEIC: Teicoplanin

An important consideration for this case is applying best practices when evaluating trauma patients presenting with LOC. While head injury is a common cause of LOC, in the absence of clear head trauma - or in the presence of subtle signs of infection- further investigation should be pursued. Our patient presented with mild fever and no overt signs of sepsis. However, the ED physicians, recognizing the atypical presentation, astutely ordered blood cultures. This decision proved critical in securing the diagnosis when the patient re-presented to the hospital.

In conclusion, *S. constellatus* is an increasingly recognized cause of pyogenic liver abscesses. When a clear portal of entry cannot be identified by physical examination or routine imaging, endoscopic evaluation should be considered to help elucidate the inciting source.

## Consent

Written consent was obtained.

## Funding

None.

## CRediT authorship contribution statement

**Yuma Takeda:** Writing – original draft. **Nicholas Van Sickels:** Writing – review & editing. **Takaaki Kobayashi:** Writing – review & editing. **Akihito Yoshida:** Writing – review & editing.

## Declaration of Competing Interest

None.
